# Outcomes of one-stage feminizing genitoplasty in children with congenital adrenal hyperplasia and severe virilization

**DOI:** 10.1007/s00383-024-05638-8

**Published:** 2024-03-06

**Authors:** Wael Abosena, Hisham AlMohamady Almetaher, Ashraf Ahmed El Attar, Ahmed Hassan Nofal, Essam Abdelaziz Elhalaby

**Affiliations:** Pediatric Surgery Department, Faculty of Medicine, Tanta University Hospital, Tanta, 31527 Egypt

**Keywords:** Feminizing genitoplasty, Congenital adrenal hyperplasia, Urogenital sinus, Severe virilization

## Abstract

**Purpose:**

To present our surgical experience and outcomes in congenital adrenal hyperplasia (CAH) patients with severe virilization using a combined technique of total urogenital mobilization (TUM) and a modified pull-through vaginoplasty to perform a safe and effective one-stage feminizing genital reconstruction for these children.

**Methods:**

Fourteen CAH patients with severe virilization, defined by a Prader IV and V rating of the external genitalia, underwent TUM followed by a limited vaginal pull-through procedure from June 2016 to December 2020. Postoperative anatomical and cosmetic outcomes, and urinary continence, were evaluated.

**Results:**

Out of the 14 cases in this study, 8 were classified as prader IV and 6 as Prader V. The median age at surgery was 11 months (range 6–36 months), and the mean urethral length was 1.4 cm (range 1.2–1.8 cm). The median follow-up period was 4 years. Our cosmetic outcomes were good in 11 (78.5%), satisfactory in 2, and poor in one case. All patients achieved age-appropriate toilet training without urinary incontinence.

**Conclusion:**

Adopting our surgical approach of TUM with modified pull-through vaginoplasty has simplified feminizing surgical reconstruction in CAH cases with severe genital atypia and a very high vaginal confluence with short urethral length, yielding adequate introitus with good anatomical and cosmetic appearance and adequate urinary continence outcomes.

**Supplementary Information:**

The online version contains supplementary material available at 10.1007/s00383-024-05638-8.

## Introduction

Disorders of sex development (DSD) encompass a spectrum of congenital conditions where there is a biological mismatch between chromosomal, gonadal, and phenotypic sex [1]. A common manifestation of DSD is ambiguous genitalia in newborns, necessitating prompt and accurate diagnosis. The most frequent cause of ambiguous genitalia is congenital adrenal hyperplasia (CAH), an autosomal recessive genetic disorder with an incidence of 1/10,000–1/15,000 live births [2, 3]. Most CAH cases are caused by mutations in the gene that encodes the 21-a-hydroxylase enzyme. This results in variable degrees of virilization due to hormonal imbalances caused by a deficiency of cortisol and aldosterone and the overproduction of androgens in the adrenal gland [4]. Depending on the extent of the enzyme defect, patients with CAH can exhibit one of three forms: the salt-wasting (SW) and simple virilizing (SV) forms, which are collectively known as classical CAH, as well as the milder nonclassical (NC) or late-onset form [5].

Girls with CAH may present with varying degrees of genital ambiguity, including a common urogenital sinus (UGS), elongation of the clitoris, and fusion of labio-scrotal folds [6]. The main goal of feminizing surgery is to separate the genital and urinary tracts, allowing for normal urination, creating a functional vaginal opening, and achieving a near-normal external genital appearance [7]. Fundamentally, the operation is dependent on the patient's anatomy, and patients are typically categorized as having either a "low confluence" or "high confluence" based on the location of the junction between the urethra and vagina [6].

From a surgical perspective, the distance between the bladder neck and the confluence is a more significant factor than the length of the common sinus. When the urethra is short and the vagina enters near the bladder neck, the surgery becomes more challenging, regardless of the length of the common sinus. This is due to the degree of mobilization required, difficult exposure, and increased risk of bladder neck or sphincter injury. However, a longer sinus can be beneficial for the surgery since it provides more tissue for repair [6, 8]. Various operations and their modifications have been reported in the literature for the management of CAH patients with high urethrovaginal confluence. In 1969, Hendren and Crawford [9] introduced a pull-through procedure, mobilizing the high vagina toward the perineum. However, the procedure posed technical challenges, primarily due to inadequate exposure and the absence of a clear dissection plane between the anterior wall of the vagina, the urethra, and the bladder neck. These factors increased the risk of injury to the urinary tract and its sphincter mechanism [10]. To improve exposure during vaginal pull-through, Salle et al. utilized the anterior sagittal transrectal approach (ASTRA) for the surgical management of the high urogenital sinus. They concluded that this technique provided excellent exposure, leading to low complication rates and facilitating the separation of urogenital sinus structures [11]. In 1997, Peña introduced the total urogenital mobilization (TUM) technique for repairing the urogenital sinus in girls with persistent cloaca, eliminating the need for separating the urethra from the vagina. The method involved en-bloc mobilization of both structures, bringing them down to the perineum to create separate openings [10]. Two years later Ludwikowski et al. used this technique for treating genital ambiguity in children with CAH via a perineal approach [12]. Subsequently, some authors raised concerns about the potential impact of TUM on urinary continence and bladder function [13]. Consequently, a procedure known as "partial mobilization of the urogenital sinus" (PUM) was introduced [14]. The purpose of our study was to report our surgical approach for performing a safe and effective one-stage feminizing genital reconstruction in patients with congenital adrenal hyperplasia who had severe Prader IV to V virilization. We hypothesized that the use of a combination technique of total urogenital mobilization (TUM) and a modified pull-through vaginoplasty is associated with favorable postoperative anatomical and cosmetic outcomes, as well as urinary continence, in these patients.

### Patients and methods

After obtaining approval from the Ethics Committee of the Tanta University Hospital, We included all CAH patients who had severe virilization (Prader IV to V) according to the Prader scoring system of the external genitalia [15]. The study period was from June 2016 to December 2020, and children who had undergone previous genital surgery were excluded.

As part of the diagnostic workup, blood electrolyte levels were measured to identify cases of salt-losing CAH, and steroid levels were measured. Chromosomal analysis was conducted to confirm the genotypic sex, while a pelviabdominal ultrasound was conducted to assess the internal sex organs. All cases underwent a retrograde genitogram to help identify the UGS anatomy and the level of vaginal confluence. A panendoscopic examination of the genitourinary tract was also carried out at the time of surgery to insert catheters into both the vagina and urethra and to verify the level of vaginal confluence and the urogenital sinus anatomy, particularly in patients whose imaging evaluation was challenging. The proximal urethral length was assessed endoscopically by introducing the cystoscope through the urogenital sinus opening. Subsequently, it was advanced proximally to the bladder neck, then carefully and slowly withdrawn to the confluence point. The length of the scope segment retracted from the external meatus on the outside was then measured, representing the urethral length.

Before the surgery, we obtained informed consent from the parents after providing them with detailed explanations of the patient's genital anatomy and the various surgical interventions available, including clitoral reduction, labioplasty, correction of the UGS, and vaginoplasty. Perioperative stress doses of steroids were given according to the endocrinologist’s instructions.

### Surgical technique

The child was positioned supine, with both legs secured to an inverted U shape bar fixed at the end of the operating table. Her genitalia and abdomen were prepped and draped in the usual standard sterile fashion. During the procedure, betadine-soaked gauze was packed into the rectum to palpate the anterior rectal wall (Supplementary Video 1).

### Clitroplasty

In all patients, we performed clitoral reduction using the Kogan technique [16], which involves subtunical excision of the two corpora while leaving the glans with the intact neurovascular bundle. A 4/0 traction suture was inserted into the clitoral glans, followed by a circular incision approximately 3–5 mm proximal to the glans. The dorsal skin was fully degloved to the base of the clitoris and then incised at the dorsal midline to form two flaps, which would be utilized to construct the labia minora and lateral vaginal wall.

We made two parallel longitudinal incisions through Buck's fascia at the lateral sides of both corpora ventrally. The midbody of the clitoris was then resected between its insertions at the base of the glans and just distal to the bifurcation of the corpora at their origins on the inferior pubic ramus. At the same time, some erectile tissue was left distal to the bifurcation to maintain a clitoral erection during sexual arousal. After completing the clitoral reduction, the clitoris was repositioned to the end of the erectile bodies using two interrupted 4-0 Vicryl sutures. The neurovascular bundles were carefully placed over the pubic bone with no tension and avoiding any kinking or trapping of the bundles. If the glans was large, a wedge resection from the ventral aspect of the glans was removed and sutured with 5/0 Vicryl.

### Vaginoplasty

**Fig. 1:**
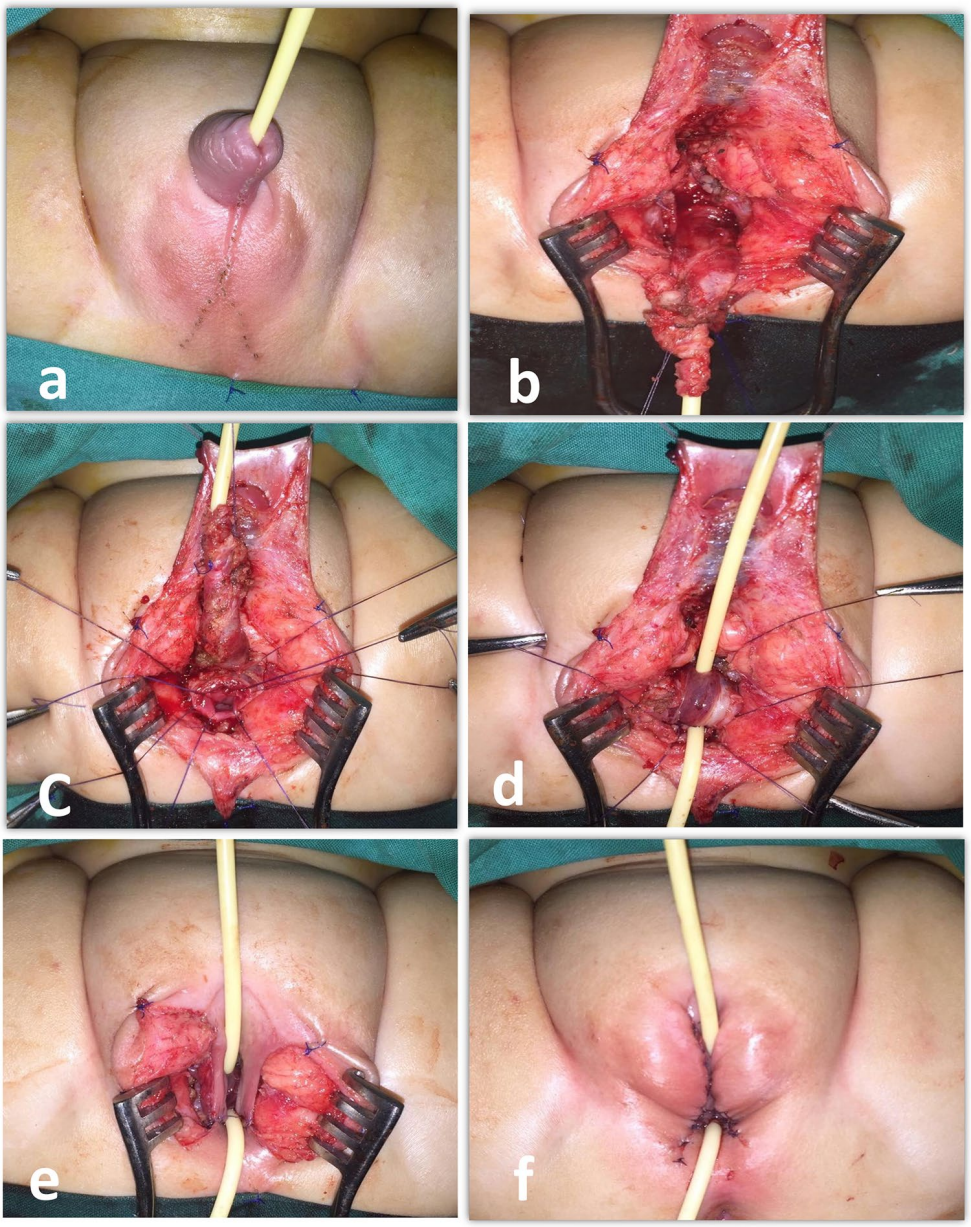
The operative technique **a** seven-month-old CAH patient marking the posterior perineal flap by fine needle diathermy. **b** TUM is completed. **c** Separating the vagina from UGS with multiple stay sutures showing the vaginal opening all around. **d** The distal part of the UGS is incised dorsally, inverted, and sutured to the anterior vaginal wall. **e** The perineal skin flap constructs the posterior vaginal wall. The preputial skin flaps construct the labia minora. **f** Clitoroplasty, vaginoplasty, and labioplasty are completed. Catheters are shown in both urethral and vaginal openings

A perineal skin flap was marked and incised in an inverted U shape, extending anteriorly to the edge of the sinus and posteriorly to the posterior margin of the new labia. The goal was to create a thick flap with ample blood supply (Fig. 1a). An incision was made around the sinus, allowing it to be mobilized circumferentially. We started with a posterior perineal dissection, creating a plane between the anterior rectal wall and the posterior urogenital sinus and vagina, extending up to the peritoneal reflection. Under direct visualization, we fully mobilized the lateral edges. Consequently, the posterior and lateral walls of the vagina and urogenital sinus were completely freed up. Next, we carefully separated the anterior wall of the UGS from the erectile bodies up to the level of the pubic bone. Then the pubourethral ligament was divided to release the UGS from the pubic bone. This caused the entire UGS to move towards the perineum as it gave away, resulting in Total Urogenital Mobilization (TUM) (Fig. 1b).

Despite undergoing TUM, the patient's vagina remained at a significant distance from the perineum, necessitating a modified (Limited) pull-through vaginoplasty to separate the urethra and vagina. However, this procedure became much easier due to the excellent exposure after the TUM. When performing the modified pull-through vaginoplasty, we identified the confluence of the vagina to the posterior urethra by applying traction on the vaginal catheter and palpating the vaginal balloon. The posterior wall of the vagina was then opened at this confluence. The anterior wall of the vagina was cautiously separated from the urethra, limited to a maximum of 0.5–1 cm for a limited pull-through procedure, without the need for extensive mobilization of the vagina. The urethral defect was repaired using 6/0 Vicryl sutures in two layers, with the mucosal edges longitudinally approximated using a running locking 6-0 Vicryl suture, followed by bringing together the lateral edges of the spongiosum as a separate layer (Fig. 1c).

The distal part of the long UGS was incised at 12 O’clock dorsally and reflected backward to reconstruct the anterior vaginal wall as described by the Passerini-Glazel technique [17] (Fig. 1d). The proximal part of UGS was used to maintain an adequate length of the urethra. The two preputial skin flaps were used to construct the lateral vaginal walls (Fig. 1e). In contrast, the posterior wall was created using the posterior perineal skin flap in all cases to create a wide vaginal introitus[18] (Fig. 1f).

### Labioplasty

Once the flaps were sutured to the vagina, the labioscrotal tissue was mobilized and trimmed. It was then moved posteriorly in a Y–V plasty fashion to create labia majora on either side of the vaginal orifice. Care was taken to preserve the subcutaneous fat necessary to give the labia their desired shape and fullness. The preputial skin flaps were used to reconstruct the labia minora. They were sutured to the lateral wall of the vagina on both sides, as well as laterally to the labia majora. After the surgery, a Foley catheter was inserted into the patient's urethra and left there for 5–7 days before removal. A vaginal pack of Vaseline gauze was used and removed after 48 h. The wound was closed by compression X-shaped sterile dressings for 48 h after surgery.

### Postoperative care

All patients were hospitalized for 7–10 days after the surgery. Steroids were continued at double the usual dose for 2–3 days post-surgery and then tapered based on the pediatric endocrinology team's instructions. Oral feeding was started within 24 h after the surgery, and suitable analgesics were given for pain management during the early recovery. Parenteral antibiotics were administered throughout catheterization.

### Follow-up visits

The follow-up schedule included one visit to the endocrinology-pediatric surgery clinic every 2 weeks for the first three months, followed by one visit per month for the next six months, and then every three months after that. During each visit, a genital examination was conducted to detect any postoperative complications. The postoperative cosmetic and anatomical results were assessed based on the criteria established by Creighton et al. [19]. Which included the symmetry of the genitals, the size and position of the clitoris, the vaginal opening, the appearance of the labia (ranging from normal to scrotalized, partial fusion, or total fusion), and the quality of the genital skin. The cosmetic outcome was categorized into three groups: good, which means the genital appearance was normal and would not be considered abnormal by someone without medical training; satisfactory, which indicates that up to two minor abnormalities were present but would not be considered abnormal by a layperson; and poor, which indicates that the genitalia appeared abnormal, with three or more abnormal features. The parents' concerns and degree of satisfaction about the cosmetic outcome regarding their children and information about the postoperative urinary continence were assessed during the follow-up visits. Incontinence was defined by the 2014 Standardization of Terminology of Lower Urinary Tract Function in Children and Adolescents [20].

## Results

Out of the 14 CAH patients in this study, the majority of patients had salt-wasting (SW) classical CAH (92.8%, 13/14), and 1 patient was simple virilizing (SV). All had a 21-hydroxylase deficiency, the most frequent genetic mutation that leads to CAH. The median age at the time of surgery was 11 months, ranging from 6 to 36 months (mean, 14.64 months; interquartile range [IQR], 11). Seven patients were less than 1 year old at surgery (7/14, 50%). The median length of follow-up was 4 years, ranging from 2 to 6 years (mean, 4.28 years; interquartile range [IQR], 1). According to the Prader classification of the external genitalia, eight patients were classified as grade IV (57.1%) and six as grade V (42.9%).

The results of both genitography and cystoscopy were consistent and provided similar information regarding the location of the vaginal confluence, indicating the level of surgical complexity in 7 out of 14 cases (50%). However, in the remaining 7 cases (50%), cystoscopy outperformed genitography in identifying the vaginal confluence and predicting the surgical difficulty. On average, the length of the urethra was 1.4 cm, ranging from 1.2 to 1.8 cm. The length of the urogenital sinus (UGS) varied between 2.6 and 3.5 cm, with a mean length of 2.9 cm.

The anatomic outcomes of the external genitalia are presented in Table 1. Among the postoperative complications, two patients experienced stenosis of the vaginal introitus, which successfully responded to repeated dilatation. Another patient had an abnormal position of the vaginal introitus, not surrounded by the labia majora, due to postoperative wound dehiscence. In addition, two patients had small clitoris, and another had absent labia minora.

**Table 1:** Anatomic outcomes following one stage feminizing genitoplasty

Total patients (no. 14)	Normal	Abnormal
Clitoris size	11	3 (2 small, 1 large)
Clitoris position	13	1 low
Vaginal introitus	12	2 small
Introitus position	13	1 not surrounded by Labia majora
Labia majora	12	2 (1 redundant, 1 scrotalized)
Labia minora	12	2 (1 poor, 1absent)

**Fig. 2:**
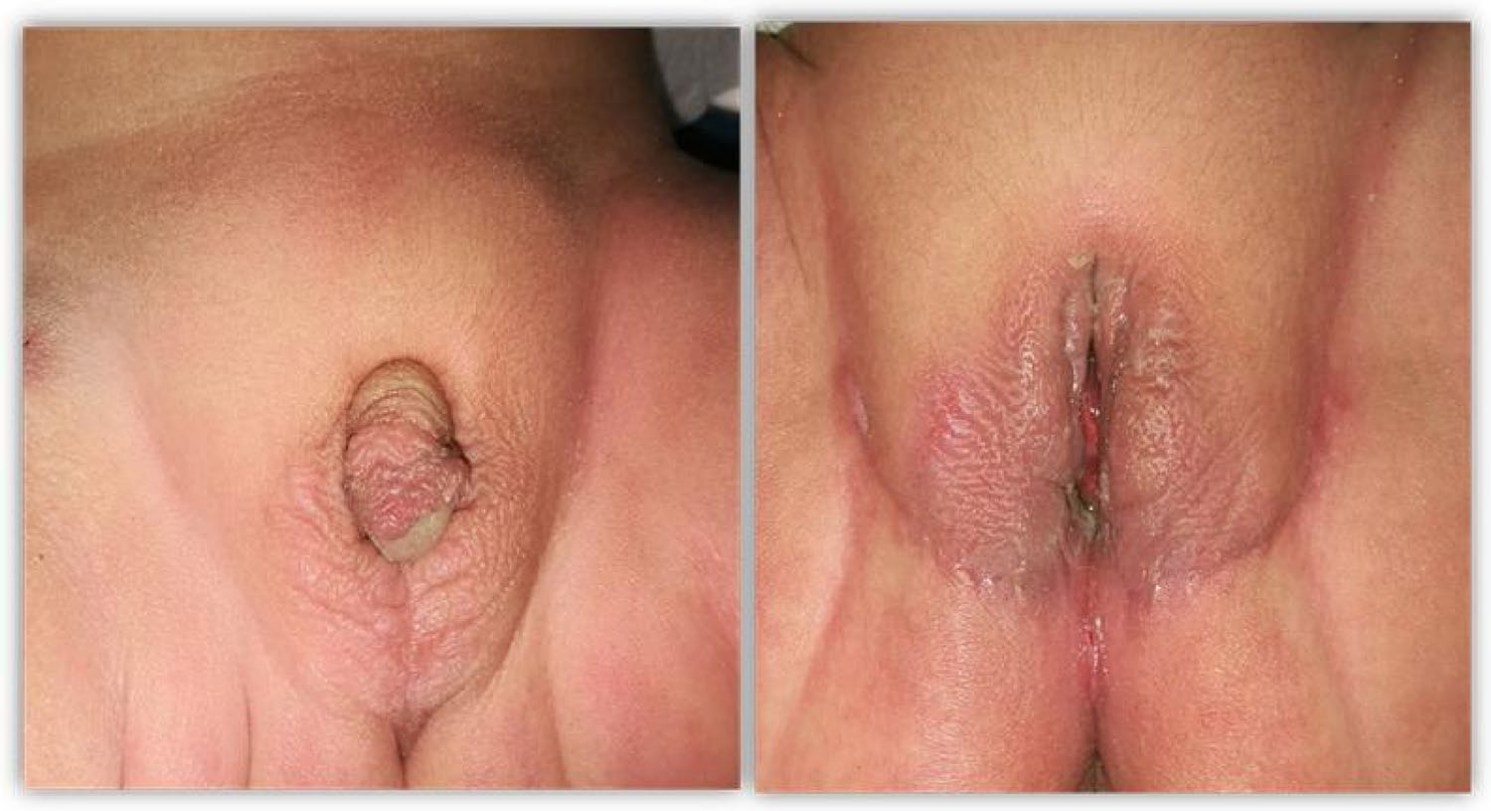
Preoperative appearance of a 6-month-old CAH patient, 4 months after one stage of feminizing genitoplasty with good anatomical and cosmetic outcomes

**Fig. 3:**
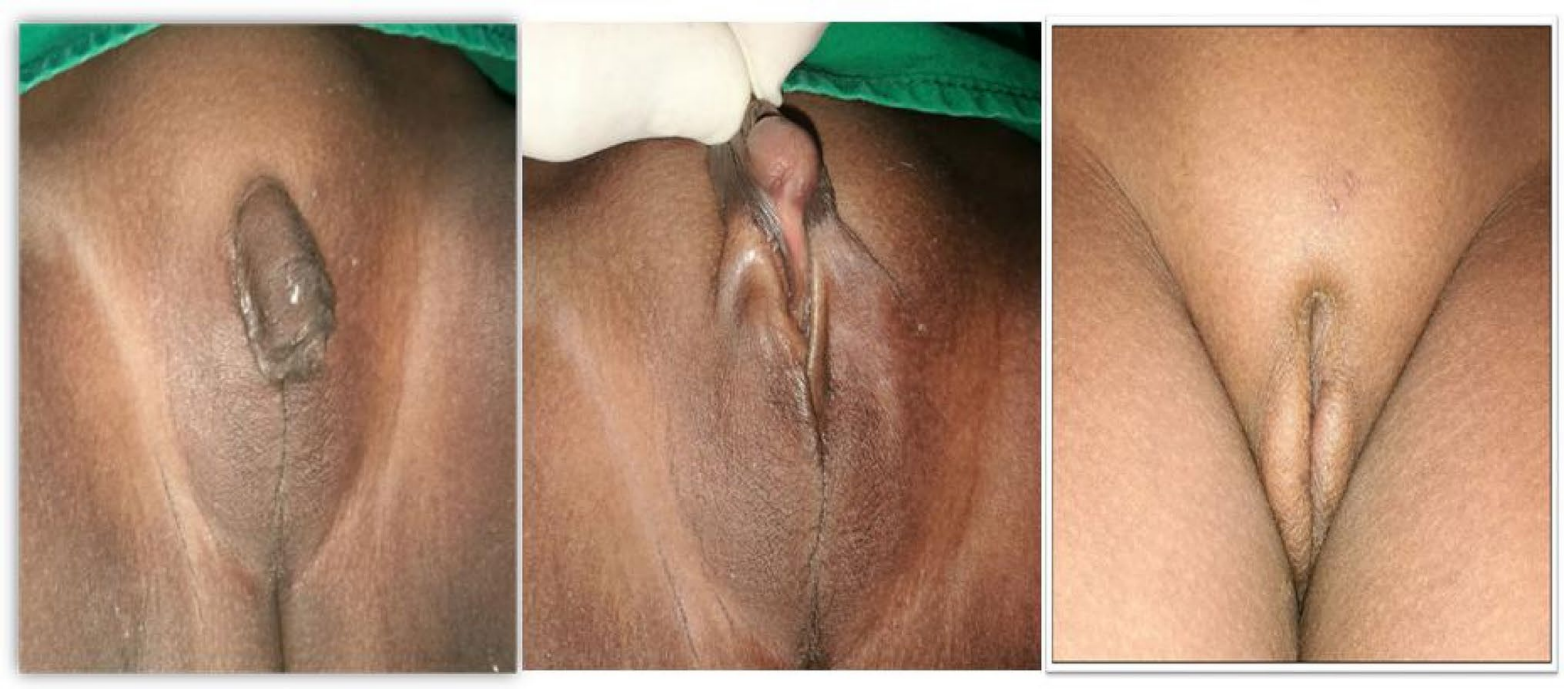
Preoperative appearance of 2.5 years old CAH patient, 6 months after one stage of feminizing genitoplasty with a good cosmetic outcome

**Fig. 4:**
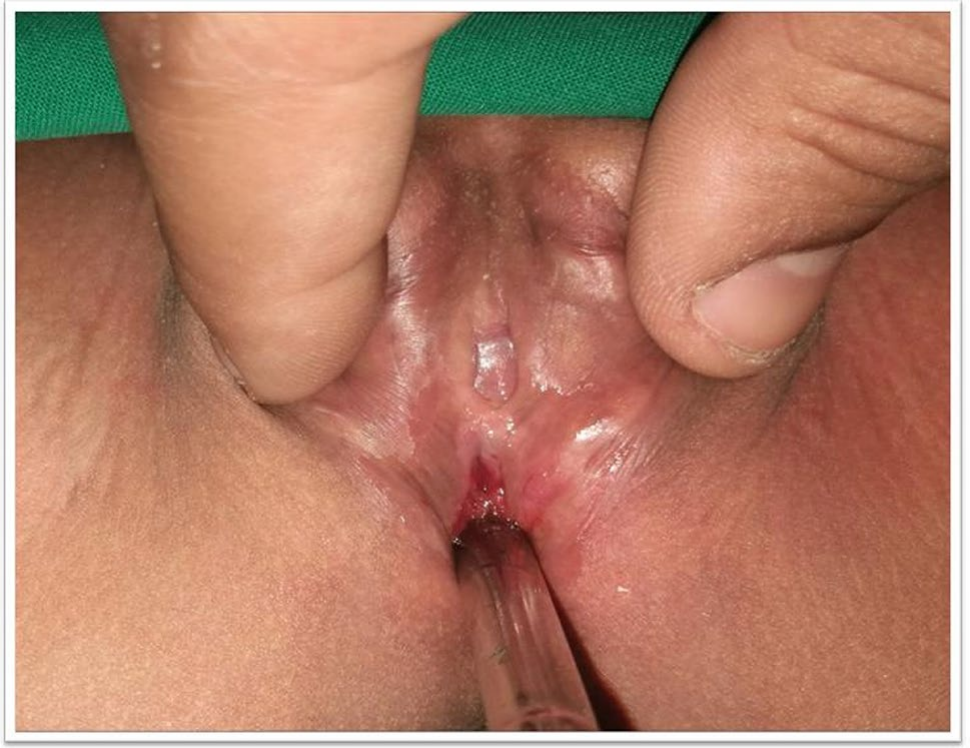
Fifteen months after a one-stage genitoplasty on a 3-year-old patient with an adequate vaginal introitus, a size 10 Hegar dilator inside, a visible anterior urethra, and a normal clitoris size and position

Regarding the overall genital cosmetic outcome of the surgery, as evaluated according to the criteria described by Creighton et al. [19] (Table 2). Eleven out of 14 cases (78.5%) exhibited a favorable good genital cosmetic appearance with adequate vaginal introitus (Figs. 2 and 3). Two patients (14.3%) yielded a satisfactory cosmetic outcome, one attributed to redundant labia majora, while the other displayed persistent suture marks and residual scrotalization of the labia majora. Unfortunately, one patient experienced a poor outcome due to a posteriorly positioned vaginal introitus, necessitating revision labioplasty. Following the surgery, all patients achieved age-appropriate toilet training, and no changes were observed in their voiding patterns during the follow-up period. All patients maintained dry intervals between spontaneous voiding and were deemed clinically continent. The urethral opening was appropriately positioned in the vestibule and easily accessible for all patients (Fig. 4).

**Table 2:** Overall cosmetic outcomes

	No (%)
Good	11 (78.5%)
Satisfactory	2 (14.3%)
Poor	1 (7.2%)

## Discussion

Reconstructive surgery for patients with CAH is usually performed to optimize external genital appearance and function while ensuring good urinary continence outcomes [21]. Over the past few decades, various surgical approaches for feminizing genital reconstruction in patients with congenital adrenal hyperplasia (CAH) have evolved. These advancements are attributed to a better understanding of female genitourinary anatomy, modifications in surgical techniques, and audit of surgical outcomes [7, 12, 14, 22–25]. In CAH patients with severe virilization (Prader IV–V), it has been widely believed that complete separation of the vagina from the urogenital sinus with a “pull-through” vaginoplasty is the optimal solution for addressing high vaginal confluence [26]. However, the procedure is technically demanding because there is no clear dissection plane between the anterior wall of the vagina, the urethra, and the bladder neck. Additionally, this area is difficult to visualize, and inadequate exposure can result in injury to the urinary tract and its sphincter mechanism. Consequently, unsatisfactory outcomes such as strictures and fistulas can occur, and often the separated vagina fails to reach the perineum [10, 27]. To enhance exposure during vaginal pull-through and separation from the urethra in cases of a long urogenital sinus, Salle et al. introduced ASTRA for the surgical management of high urogenital sinus. The study, involving 16 patients with a high urogenital sinus secondary to CAH, demonstrated the excellence of this technique, showing low complication rates and improved separation of urogenital sinus structures. All patients maintained continence post-surgery, although one patient developed sepsis, requiring a second surgical procedure. Despite the benefits of low incontinence risk and improved exposure, ASTRA is considered a more aggressive approach, involving rectal opening and greater complexity due to the need for a change in patient position [11]. The current findings demonstrate the feasibility of separating the vagina from the urethra via the perineal route, even in cases of high urethrovaginal confluence, without resorting to ASTRA [7, 28].

The TUM technique, as described by Pena, offers the advantage of avoiding the need for dissection to separate the vagina from the urethra. Instead, both structures are mobilized and brought together to the perineum [10]. Subsequently, Ludwikowski et al. used this technique for treating genital ambiguity in children via a perineal approach [12]. However, in children with the highest-level confluences (Prader IV–V) and a very short urethral length proximal to the confluence of the vagina and UGS, the TUM technique cannot be used alone due to a high risk of urinary incontinence and even after TUM, the distance between the vagina and perineum remains significant [7, 12, 14]. To address these issues in our series, we used the technique of combined TUM and modification of the Hendren and Crawford pull-through vaginoplasty (limited pull-through vaginoplasty) [9].

Our surgical technique combines modifications and concepts of previously reported feminizing genitoplasty procedures to achieve the best outcome [17, 25]. The surgical technique of modified Hendren and Crawford pull-through vaginoplasty is not new, but it was first described by Gonzalez and Fernandes [25]. However, their study used the prepuce to construct the vestibule and anterior vaginal wall, while the UGS was left as a urethra. In our series, combining TUM and the modified Pull-through procedure has simplified surgical reconstruction for severely virilized CAH patients, resulting in good postoperative outcomes and urinary continence. Our approach offers several advantages, including excellent exposure, utilization of mobilized UGS tissue to create a vestibule with a lining of mucosal tissue, and reconstruction of the anterior vaginal wall and urethra. Moreover, using the distal UGS tissue to construct the anterior vaginal wall by reflecting it backward provides excellent coverage in the region of the vaginal separation from the urethra, which helps in preventing the formation of a urethrovaginal fistula.

Importantly, our approach avoids the necessity of completely separating the vagina from the urethra and bladder neck, which carries a significant risk of complications and injury to the urinary tract. In our surgical experience, we reported good to satisfactory genital cosmetic outcomes (92.8%, 13/14), as judged by both the surgical team and our children's parents. The urethral meatus was orthotopic and easily accessible in all patients. In two of our early cases, mild vaginal stenosis developed postoperatively, but it successfully responded to repeated dilatation. All patients in our study had age-appropriate toilet training and achieved urinary continence.

Concerns have been raised in the medical literature regarding the potential development of urinary incontinence after TUM due to the dissection of the anterior urethra from the pubourethral ligaments. Stites et al. reported two cases of urinary incontinence among ten CAH cases corrected by TUM due to extensive dissection of the anterior urethra from the pubourethral ligaments [29]. To address this concern, Rink et al. developed a modified technique called partial urogenital mobilization (PUM). This technique involves the same circumferential dissection as TUM, but it stops at the level of the pubourethral ligament [14]. However, in children with the highest level of vaginal confluences (Prader IV–V), as in our series, PUM was not enough for surgical correction of the UGS. Even after TUM, the distance between the vagina and the perineum remained significant [30]. Nevertheless, multiple investigators reported the safety of TUM and have demonstrated good urinary continence outcomes [7, 31–34]. Kryger and González conducted a study on 13 girls who underwent TUM and utilized non-invasive urodynamics and a questionnaire. The findings revealed no cases of incontinence attributable to the operation [31]. Similarly, Braga et al. reported on 10 girls with CAH who were monitored for 26 months after distal TUM, and they observed no evidence of incontinence or vaginal stenosis [35]. Furthermore, Camanni et al. followed six girls with CAH who underwent TUM correction and found no instances of bladder dysfunction or incontinence [36]. A review of the urology literature highlighted the importance of urethral length in decision-making and surgical planning for the repair of a urogenital sinus, with or without congenital adrenal hyperplasia [37, 38]. Improved patient selection for TUM based on urethral length could potentially reduce urological morbidity [39]. The literature suggests that a urethral length greater than 1.5 cm is necessary for the success of TUM surgery in patients with a urogenital sinus [33, 39]. Adopting our combined TUM and limited vaginal pull-through technique has yielded several advantages resulting in favorable postoperative cosmetic and urinary continence outcomes. Firstly, we successfully maintained an adequate urethral length of more than 1.5 cm by preserving the proximal part of the UG sinus to lengthen the urethra and maximize the opportunity for future urinary continence [40]. Secondly, we avoided the complete separation of the vagina from the urethra and bladder neck, a measure taken to mitigate the significant risk of bladder neck injury and potential damage to urethral innervation [41]. Thirdly, during TUM, we exercised caution by refraining from aggressive anterior proximal urethral dissection post-pubourethral ligament division. Additionally, we did not divide the endopelvic fascia to prevent bladder neck injury or caudal displacement, which could impact the continence mechanism [33]. Finally, our approach provided excellent exposure, simplifying surgical reconstruction for our severely virilized CAH patients.

Traditionally, genitography and endoscopy have been used to evaluate the genitourinary tract, identify the level of the vaginal confluence, and delineate the anatomy of the UG sinus. However, our study found that genitography was less accurate in detecting vaginal confluence in 50% of cases. Following endoscopic assessment of these patients, we identified a highly narrow vaginal orifice hidden within the mucosal folds of the pseudo verumontanum. This may explain the challenges in contrast passage through the vaginal opening, leading to difficulties in delineating the site of communication between the vagina and urogenital sinus and subsequently resulting in a less accurate assessment of the urethral length proximal to the confluence. Donahoe and Gustafson have also questioned the reliability of genitography, as a high confluence may not be identifiable radiographically. Instead, careful endoscopy might be necessary to identify a narrow vaginal orifice concealed within the mucosal folds of the pseudo verumontanum [42]. Podesta and Urcullo shared a similar perspective, suggesting that combining both investigations provides the most comprehensive understanding of confluence depth and the proximal urethra [43].

Despite surgical approaches to genital reconstruction in CAH patients being performed for decades, the age at which surgery is best conducted, which type of procedure offers the best outcome, and whether surgery should be performed in all patients continues to be intensely debated. Proponents of early surgery argue that the procedures are easier to perform and lead to better outcomes in young children. They also assert that operating in infancy reduces the stigma associated with living with genital ambiguity, both for the patient and their family [25, 44]. Lobe et al. reported better outcomes in patients diagnosed and operated on during infancy [45]. Similarly, Passerini-Galazel stated that surgery could be easily performed within the first 1–2 months of life, but revision during puberty should be anticipated in some cases [17]. According to the 2006 Consensus Statement, feminizing genitoplasty should be considered in girls with severe virilization (Prader III–V) within the first year of life in specialized centers [46]. In our study, 50% of our patients were operated on before the age of one year, ranging from 6 to 10 months. We noted that the surgical technique was much easier in patients under 1 year of age, and the vaginal tissue was easy to handle and not thin or fragile compared to older children. In our community, this type of surgery is embarrassing for both patients and parents, and the parents highly appreciate early reconstruction during infancy. The psychological benefits of early vaginoplasty should be weighed against the potential need for some revision after puberty, particularly in patients with CAH and severe virilization that result in abnormal-appearing genitalia [47]. According to Lean et al., a planned one-stage feminizing genital reconstruction results in better outcomes than multistage genital surgery, with an expected 88% incidence of good cosmetic results. Repeated surgeries can lead to more scarring and fibrosis. Clitoroplasty, labioscrotal reduction, and exteriorization of the vagina should be done together to take advantage of all available tissue [48, 49]. Our series achieved 78.5% good and 14.3% satisfactory cosmetic outcomes with one-stage early genital surgery in CAH patients with severe virilization. This indicates that one-stage feminizing surgery can be performed early with good results, provided that the surgeon is familiar with the procedure.

While our study is limited by small sample size and a lack of long-term follow-up, we believe that our exclusive focus on feminizing genital reconstruction using a combined technique of TUM and modified pull-through vaginoplasty for CAH patients with severe virilization is a strong point. Furthermore, our small sample size represents a clinically significant group in the context of the existing literature on surgical reconstruction and postoperative outcomes of CAH patients with severe genital atypia.

## Conclusion

Feminizing genital reconstruction can be performed safely and effectively in severely virilized CAH patients, resulting in adequate introitus with excellent cosmetic appearance and good anatomical and adequate urinary continence outcomes. The procedure can be done in one stage during infancy with favorable outcomes, provided that the surgeon is familiar with the procedure. Adopting our combined TUM technique and modified pull-through vaginoplasty has simplified the surgical reconstruction and improved outcomes in CAH cases with severe genital atypia with a very high vaginal confluence and a short urethral length.

## Supplementary Information

Below is the link to the electronic supplementary material.

Supplementary file1 (MP4 63678 KB)

## Data Availability

The data supporting the findings of this study are available from the corresponding author upon reasonable request.
